# Gauging force by tapping tendons

**DOI:** 10.1038/s41467-018-03797-6

**Published:** 2018-04-23

**Authors:** Jack A. Martin, Scott C. E. Brandon, Emily M. Keuler, James R. Hermus, Alexander C. Ehlers, Daniel J. Segalman, Matthew S. Allen, Darryl G. Thelen

**Affiliations:** 1 Department of Materials Science and Engineering, University of Wisconsin-Madison, Madison, WI 53706 USA; 2 Department of Mechanical Engineering, University of Wisconsin-Madison, Madison, WI 53706 USA; 3 School of Engineering, University of Guelph, Guelph, ON N1G 2W1 Canada; 4 Department of Biomedical Engineering, University of Wisconsin-Madison, Madison, WI 53706 USA; 5 Engineering Physics Department, University of Wisconsin-Madison, Madison, WI 53706 USA; 6 Department of Mechanical Engineering, Michigan State University, East Lansing, MI 48824 USA; 7 Department of Orthopedics and Rehabilitation, University of Wisconsin-Madison, Madison, WI 53706 USA

## Abstract

Muscles are the actuators that drive human movement. However, despite many decades of work, we still cannot readily assess the forces that muscles transmit during human movement. Direct measurements of muscle–tendon loads are invasive and modeling approaches require many assumptions. Here, we introduce a non-invasive approach to assess tendon loads by tracking vibrational behavior. We first show that the speed of shear wave propagation in tendon increases with the square root of axial stress. We then introduce a remarkably simple shear wave tensiometer that uses micron-scale taps and skin-mounted accelerometers to track tendon wave speeds in vivo. Tendon wave speeds are shown to modulate in phase with active joint torques during isometric exertions, walking, and running. The capacity to non-invasively assess muscle–tendon loading can provide new insights into the motor control and biomechanics underlying movement, and could lead to enhanced clinical treatment of musculoskeletal injuries and diseases.

## Abstract (web summary)

Mechanical forces exerted on tendons during locomotion cannot be readily measured without invasive methods. Here, the authors develop a non-invasive wearable device to track tendon loads by measuring shear wave propagation speed, and demonstrate its use during dynamic human movements.

## Introduction

There has long been a fascination with understanding the muscle forces that actuate human movement. In the seventeenth century, Giovanni Borelli analyzed muscle lever systems and deduced that the muscle forces acting within the body must be considerably larger than the external forces acting on the body^1^. Today, we can easily measure external forces during movement. However, we still cannot readily assess the underlying muscle–tendon forces that generate human movement. Animal studies have used surgically implanted force sensors to gain insights into the control and mechanics of muscle–tendon units. However, the invasiveness of implanted sensors limits their usefulness for human studies^2–4^.

There have been prior attempts to non-invasively track muscle and tendon forces based on wave propagation measures. For example, it has been shown that the speed of axial sound waves (compression waves) in tendon varies with loading during movement^5^. Sound wave propagation is governed by tissue elasticity though, such that sound wave speed primarily provides a metric of tissue stiffness rather than force^5^. Shear wave speed has also been shown to increase with load in muscle^6–9^ and tendon^10,11^. However, this dependency is generally attributed to a strain-stiffening increase in the tissue’s shear modulus^12^. Further, current ultrasonic shear wave measurement systems lack the range and temporal resolution for tracking tissue wave speeds during dynamic movement.

This work introduces a new approach to track and interpret tendon shear wave speeds during dynamic movement. We first present a tensioned beam model and ex vivo measurements showing that shear wave propagation speed in tendon depends primarily on axial stress at physiological loads. This phenomenon is akin to the tension-dependent vibration frequencies of guitar strings. We then show that in vivo tendon wave speeds can be dynamically tracked using a remarkably simple shear wave tensiometer, consisting of a skin-mounted tapping device and miniature accelerometers. Lower extremity tendon wave speeds are shown to vary closely with joint kinetics during active exertions, and also detect phases of passive tendon tensioning which have previously only been detected with invasive sensors^13^. We envision numerous uses of the technology for fundamentally investigating the motor control and tissue mechanics underlying normal movement. This technical advance can also address many important applications spanning orthopedics, rehabilitation, ergonomics, and sports.

## Results

### Model of shear wave propagation in a tensioned tendon

We model tendon as a tensioned Timoshenko beam^14^ exhibiting locally linear elastic behavior. At high vibration frequencies, shear deformation is dominant and the equation of motion for incremental transverse deflection, *w*, is:

1 $${\it{\rho }}\frac{{\partial ^2w}}{{\partial t^2}} = \left( {k{\prime}{\it{\mu }} + {\it{\sigma }}} \right)\frac{{\partial ^2w}}{{\partial x^2}},$$

where *ρ* is the effective density of the material, *k’* ( < 1.0) is the shear correction factor^15^ which accounts for the shape of the cross-section, and *x* is the axial position along the beam (see supplementary methods for details). Assuming that transverse deflection is harmonic in time and space, the shear wave speed, *c*, is a function of the shear modulus, *μ*, of the tissue and axial stress, *σ*, acting on the transverse cross-section of the tendon:

2 $$c^2 = \frac{{k{\prime}{\it{\mu }} + {\it{\sigma }}}}{{\it{\rho }}}.$$

The derivation is based on incremental motion about a stressed state, so the shear modulus term, *μ*, represents the tangential modulus for tendon which exhibits nonlinear material behavior^16,17^. Additionally, since this relationship is derived based on Timoshenko beam theory^14^, *μ* can readily accommodate the transversely isotropic material behavior exhibited by tendon^18,19^. The co-dependence of wave speed on shear modulus and stress is also seen in acoustoelastic wave propagation models that explicitly account for external loading and its effects on nonlinear material properties^20^. Most shear wave studies to date make use of the special case in which the axial stress is zero and the shear correction factor is unity, the latter of which occurs if the finite nature of the cross-section is neglected. In this case, a planar shear wave obeys the wave speed equation $\left( {c^2 = {{\it{\mu }}/{\it{\rho }}} } \right)$ that has been used to estimate shear modulus from wave speed in many soft tissues^21^, including muscle^22^ and tendon^10^. However, we hypothesize that the direct effect of stress on tendon wave speed can become dominant during movement. For example, the Achilles tendon transmits axial stresses greater than 20 MPa during walking^4^, and exceeding 50 MPa during more dynamic movements^3,13^. In contrast, the shear moduli of ligaments, which have similar hierarchical structure to tendon, range from 0.04 to 1.6 MPa under shear deformation^16,17^. Thus, the tangential shear modulus term, which can account for nonlinear strain-stiffening, is predicted to have negligible effects on wave speed when compared with the axial stress term. This leads to the prediction that squared wave speed should vary in proportion to tendon stress $\left( {c^2 \propto {\it{\sigma }}} \right)$ under physiological loads.

To test this prediction, we first simulated shear wave propagation in a nonlinear finite element model of tendon. A transversely isotropic hyperelastic material model was used to describe the behavior of aligned collagen fibers embedded within an isotropic matrix^18^. The model-predicted wave speeds agreed very closely with the tensioned beam model over a plausible range of shear moduli and axial stress (Supplementary Fig. 1). The model also supports the assertions that the influence of the tangent shear modulus on wave speed diminishes as axial stress increases.

### Ex vivo measurement of tendon vibration

**Fig. 1:**
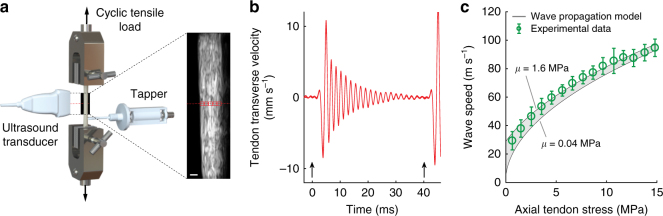
Ex vivo experiment. **a**. Porcine tendons were clamped and cyclically loaded up to 300 N. A tapper device (Supplementary Fig. 2) delivered an impulsive 20 μm tap in the transverse direction at 40 ms intervals. Ultrasound radiofrequency data were collected at 14,100 frames per second from a single location along the tendon and used to track transverse tendon displacements. Scale bar, 2 mm. **b** An underdamped standing wave emerged in response to each tap (indicated by arrows) and was used to ascertain the natural frequency and corresponding shear wave speed. **c** Plotted are the mean (±1 s.d.) wave speeds versus the corresponding tendon stress for 10 porcine digital flexor tendons. The shaded region reflects the wave speed predictions using the wave propagation model (Eq. 2) with the following parameters: *ρ* = 1730 kg m^−3^; *μ* = 0.04–1.6 MPa; *k’* = 0.9 (see Supplementary Methods, Supplementary Tables 1 and 2)

We subsequently conducted a set of ex vivo studies to determine whether the analytical model would hold in isolated tendons (Fig. 1). Porcine digital flexor tendons (*N* = 10) were cyclically loaded up to 300 N at 0.5, 1.0, and 2.0 Hz. Micron-scale tendon vibrations were intermittently induced using a piezo-actuated tapper (Supplementary Fig. 2) and recorded via high frame rate ultrasound. Each tap induced an underdamped standing wave with a frequency that increased with tensile loading (Supplementary Fig. 3). Shear wave speed, *c*, was computed (*c *= 2*fL*) from the vibration frequency, *f*, and the grip-to-grip length, *L*, of the tendon. As predicted by the tensioned beam model, wave speed monotonically increased with the square root of axial stress. Further, squared wave speed was highly correlated with axial tendon stress, with coefficient of determination (*R*^2^) of 0.96 ± 0.04 (mean ± s.d.) across the 10 tendons tested. This linear relation between stress and squared wave speed was independent of loading rate (*p* = 0.73).

### In vivo assessment of tendon shear wave speed

**Fig. 2:**
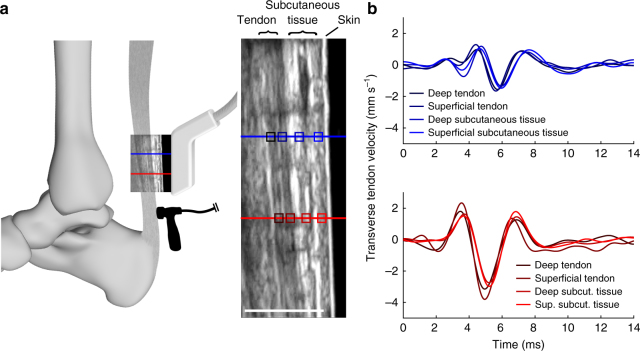
In vivo ultrasonic measurement of wave speed. **a** A linear array ultrasound transducer was strapped over the subject’s Achilles tendon. High frame rate (14,100 frames per second) ultrasonic radiofrequency (RF) data were collected at two points (red, blue lines). A custom tapper device (hammer icon; see Supplementary Fig. 2), which was located distal to the ultrasound transducer, intermittently induced transverse waves in the tendon. Center: transverse tissue velocity was measured at multiple kernels (indicated by boxes; darker shades indicate greater depth) located along each RF collection line (red, blue). Scale bar, 1 cm. **b** We observed that transverse motion was similar for all kernels along the first (red, lower) and second (blue, upper) measurement locations. Thus, tendon wave motion could conceivably be measured from the motion of subcutaneous tissue

Buoyed by the ex vivo results, we explored whether we could induce and track shear waves speeds in vivo in human tendons. We constructed a piezo-actuated tapper (Supplementary Fig. 2) that could deliver micron-scale impulses through the skin to superficial tendons. With the tapper secured over the Achilles tendon, the induced tendon motion could be felt with the fingertips up to a few centimeters away. High frame rate (14,100 frames per second) ultrasonic collections were then used to measure the wave arrival times at two points along the tendon (Fig. 2). Unlike the standing waves seen ex vivo, each in vivo tap induced a transient wave that propagated along the tendon and then dissipated into the adjacent tissue. In this case, wave speed could be computed knowing the distance between elements and the difference in wave arrival times, as is commonly done with transient shear wave elastography^21,23^.

**Fig. 3:**
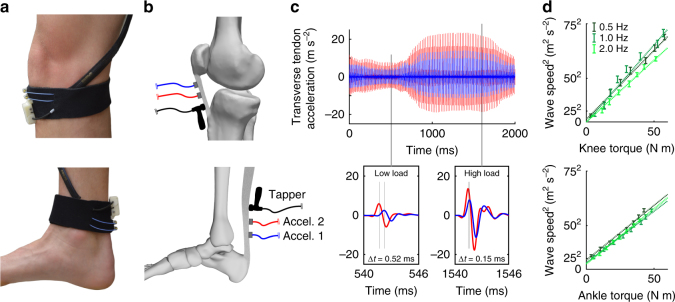
In vivo wave speed measurements. **a** Shear wave tensiometers secured over the patellar and Achilles tendons. **b** The tensiometers consisted of a piezo-actuated tapper and two miniature accelerometers spaced a fixed distance apart. **c** Representative recording of patellar tendon transverse accelerations demonstrate that the travel time (Δ*t*) between accelerometers is shorter in the high load condition, reflecting faster wave propagation. **d** Linear relationships between joint torque and squared wave speed (binned means ± 1 s.d. for a representative subject) were observed in both tendons across a range of loading rates

We were surprised to see in our initial in vivo experiments that both the tendon and adjacent subcutaneous tissue exhibited similar transient motion as a wave passed by (Fig. 2). This observation, supported by prior surface wave studies in muscle^24^ and tendon^25^, suggested that tendon shear wave vibrations may be detectable via skin-mounted sensors. To further explore this idea, we constructed tendon tensiometers consisting of a tapper and two adjacent miniature accelerometers housed in an elastic strap (Fig. 3a, b). The tensiometers were secured superficial to the patellar and Achilles tendons of five subjects, and taps were delivered at 50 Hz. Each tap induced a transient wave that could be detected in succession by the near and far accelerometers. We found that the time interval between wave arrival at the two sensors was clearly modulated by load applied to the tendon (Fig. 3c), with time lag decreasing as the tendon was tensioned.

During cyclic isometric knee extension and ankle plantarflexion, wave speeds varied in sync with the net joint torques, ranging from 15 to 20 m s^−1^ when the muscles were relaxed, up to 60–80 m s^−1^ when maximal contractions were performed (Supplementary Fig. 4). Under simple isometric loading conditions, it can be assumed that antagonist muscles are relaxed and tendon moment arms are relatively constant. Therefore, the tensioned beam model (Eq. 2) predicts that wave speed in agonist tendons should be directly related to joint torque. Indeed, linear correlations between squared wave speed and joint torque were strong for both the Achilles (*R*^2^ *=* 0.96 ± 0.04, mean ± s.d.) and patellar (*R*^2^ *=* 0.98 ± 0.01) tendons. These results verified that our non-invasive tensiometer devices could be used to infer loading of superficial tendons during static activities.

**Fig. 4:**
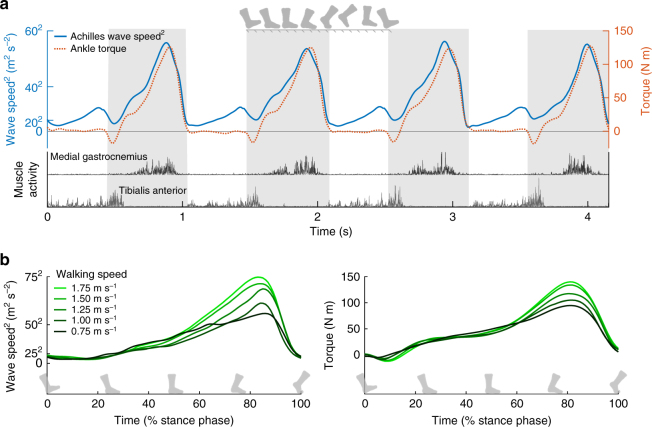
In vivo gait experiment. **a** Achilles tendon wave speed, ankle plantarflexion torque, and medical gastrocnemius and tibialis anterior muscle activity over 4 consecutive strides of treadmill walking at 1.5 m s^−1^. The primary peak in wave speed corresponds to push-off in late stance (stance indicated by shaded regions), when the gastrocnemius is active and ankle torque is high. However, a secondary peak in wave speed is seen in late swing and likely reflects passive tendon stretch due to antagonistic tibialis anterior activity. **b** Stance phase plots, ensemble averaged over multiple gait cycles for a representative subject show speed-related modulation of both Achilles tendon wave speed and ankle plantarflexion torque

The ultimate value of the shear wave tensiometers is to assess loads acting on individual muscle–tendon units during movement. To begin to assess this functionality, we tracked Achilles tendon wave speeds during treadmill walking in five subjects while simultaneously measuring joint kinetics and muscle electromyographic (EMG) activities. We found that the tapper-induced Achilles tendon accelerations were detectable throughout the walking gait cycles (Supplementary Fig. 5), and could be used to track dynamically varying wave speeds. Achilles tendon wave speeds were lowest during early swing, and peaked to a maximum of 50 to 80 m s^−1^ when plantarflexor muscles were active at push-off. During stance, wave speeds varied in phase with ankle torque and exhibited walking speed-dependent modulation (Fig. 4, Supplementary Fig. 6). Squared wave speed profiles are generally consistent with direct tendon load measures previously ascertained using invasive sensors^4^. However, the Achilles tendon shear waves do somewhat differ from prior measures of compression wave profiles in the same tendon, which exhibit a less distinct peak during push-off^26,27^. This is not surprising, given that compression wave speeds are determined by the tangential elastic modulus^26^, which varies nonlinearly and becomes increasingly less sensitive to force when a tendon is taut^19,28,29^. Interestingly, shear wave speed measures could detect tendon loading that is not captured by conventional joint torque or muscle activity measures. Just before the heel hits the ground in late swing, the primary plantarflexors (gastrocnemius and soleus) are inactive and net ankle torque is near zero. Thus, conventional motion capture and EMG data may lead one to assume that the Achilles tendon force is near zero. However, a distinct increase in Achilles tendon wave speed is seen just before heel strike (Fig. 4a), and likely reflects passive stretch of the Achilles tendon prior to impact. This observation agrees with tendon loading data collected using invasive sensors^4^ and passive tendon stretch measured with cine ultrasound imaging^30^.

### Applications of tendon shear wave metrics

**Fig. 5:**
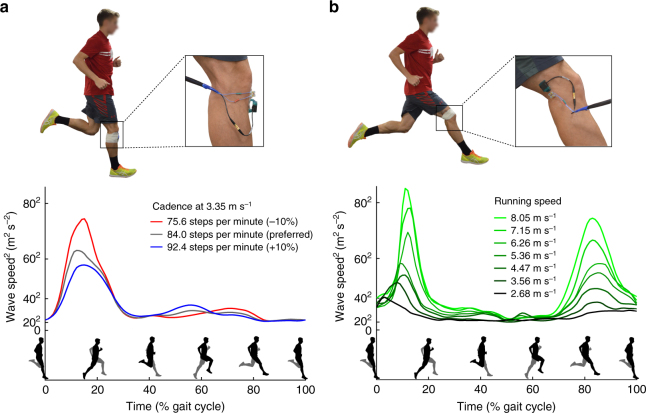
Running experiments. **a** Patellar tendon wave speeds speed over the gait cycle when running at a fixed speed with different step rates. **b** Lateral hamstring (biceps femoris) tendon wave speeds detect utilization of this muscle with increasing speed during both mid-stance (15% of gait cycle), and late swing (85% of gait cycle). Hamstring loading during both phases increased markedly with speed, though stance phase demands greater tissue loads than swing phase in this individual

We envision both clinical and fundamental uses of shear wave tensiometer technology. As a clinical example, shear wave speed measures could be used to assess gait retraining in sports medicine, physical therapy, and neurorehabilition settings. For example, step rate retraining is advocated for treating anterior knee pain in runners^31^. To assess the intervention, we measured wave speeds in the patellar tendon while a subject ran at a fixed speed (3.35 m s^−1^) with different step rates (Fig. 5a). Wave speed patterns were consistent with knee extension torques during stance, and showed similar step rate dependence^31^. For this subject, the wave speed data suggest that a +10% increase in step rate from preferred could reduce patellar tendon stress by 18%. Such information could conceivably be used as biofeedback to guide gait retraining, and to track tissue load changes with injury and treatment.

A more fundamental use of tendon wave speed data would be to investigate how the nervous system coordinates synergistic muscles during movement. There are many more muscles than joint degrees of freedom in the human body, leading to an indeterminate relationship between muscle forces and joint moments^12^. Mathematical solutions to this indeterminacy problem have been used for over 30 years^32,33^, but lack the human data needed for rigorous evaluation^12^. For this example, we used wave speed metrics to assess biceps femoris loading at speeds ranging from a slow jog (2.68 m s^−1^) to a fast sprint (8.05 m s^−1^) (Fig. 5b). The direct wave speed measurements show that the biceps femoris tendon loading is bimodal over the gait cycle, with distinct bursts in late swing and early stance. While loading in both phases clearly increased with speed, the stance phase induced ~26% higher hamstring load than the swing phase at the fastest speed. Such objective data could address long-standing debates about the coordination of synergistic muscles^12^ and function of biarticular muscles^34^.

## Discussion

There are two salient findings from this study that should enable the use of shear wave measures for investigations of tissue loads during human movement. First, we showed via models and ex vivo experiments that shear wave speed depends directly on tendon stress at functional loads, whereas prior studies have only considered low-load behavior where the tissue’s shear modulus has a more pronounced effect^10,35,36^ (Supplementary Fig. 1). The direct dependence on tension provides a fundamental basis to use wave speed as a sensor of loading. Second, we showed that micron-scale vibrations can be induced externally in superficial tendons and detected with skin-mounted accelerometers during dynamic movements. This finding led to the design of a simple tendon tensiometer that could be easily deployed in laboratory, clinic, workplace, and athletic environments.

We found a strong linear relationship between squared wave speed and joint torques during simple isometric exertions, providing confidence in using the devices to track in vivo tissue loads. Achilles tendon wave speed measures during gait were aligned with active joint torques, while also revealing phases of passive loading that are not evident in traditional joint kinetic measures. Further study is needed to investigate how one can estimate absolute tendon tension from in vivo shear wave speed metrics. When shear modulus is low relative to stress, our analytical model suggests that one could estimate tension, *T *≈ *Aρc*^2^, from wave speed (*c*) knowing the effective tissue density (*ρ*) and cross-sectional area (*A*). Tendon cross-section is relatively easy to assess using either ultrasound or magnetic resonance imaging protocols, but effective density is more challenging. For our ex vivo study, we estimated the effective density by modeling the beam as an elliptical cross-section vibrating in water. Clearly, the in vivo case is more complex with the potential for tendon vibration to induce motion in adjacent subcutaneous tissue and muscle. We are embarking on a set of in situ validation experiments in which tendon wave speeds and tension are monitored simultaneously during robotic gait simulations^37^. These experiments will be used to assess inter-tendon variability in the wave speed–stress relationship, which may be attributable to differences in tendon geometry or added mass effects associated with adjacent tissues.

If inter-tendon variability in shear wave speed metrics cannot be well explained by morphological measures, it is feasible to envision a simple calibration procedure. Simultaneous recordings of shear wave speed and joint torques can be made during isometric exertions (Supplementary Fig. 4). Tendon tension can be estimated from joint torque using imaging-based measures of the tendon’s moment arm^38^ and assumptions on muscle load sharing among both agonist and antagonist muscles^32^. A squared wave speed–tension calibration can then be attained. Subsequently, shear wave speeds measured during complex dynamic movement could be used to estimate absolute tendon tension. Our approach for estimating tension from shear wave speed is premised on the assumption that the shear modulus is relatively low under physiological loads, similar to shear modulus values observed in ligaments subjected to shear deformation^16^. Additional studies are needed to directly probe the relationship between tendon shear modulus and tension under axial loading conditions. Further, our tensioned beam model describes plane wave behavior. More complex wave propagation models based on acoustoelastic theory^20^ may be necessary to understand variations in shear wave speed over the tendon cross-section, which could arise due to heterogeneity or localized tissue damage.

In summary, we have introduced a coupled theoretical and practical implementation for using wave speeds to gauge tendon loading in superficial tendons during dynamic movement. The tensiometers we introduce utilize miniature accelerometers as the primary sensor modality, making the technology relatively low cost and potentially usable in field environments. We envision numerous applications for investigating the motor control underlying normal movement and for identifying the biomechanical factors contributing to movement disorders. Further, muscle–tendon forces induce much of the loading and deformation that occurs in ligament, cartilage, and skeletal tissues^39^. Thus, muscle–tendon load information could be used to guide rehabilitative interventions, plan orthopedic procedures, assess tissue healing following treatment, monitor activity, and engineer viable tissues that can restore lost or impaired motor function.

## Methods

### Finite element model

Tendon behavior was described by an incompressible, transversely isotropic hyperelastic material. The structurally motivated continuum material model presumes the elastic response of the tissue to arise from aligned collagen fibers embedded in an isotropic matrix. The strain energy density function *W* contained three terms representing the matrix, collagen fibers, and tissue volumetric response given by^18^:

3 $$W = F_1\left( {\tilde I_1,\tilde I_2} \right) + F_2\left( {\tilde \lambda } \right) + \frac{K}{2}\left( {\ln \;J} \right)^2,$$

where $\tilde I_1$ and $\tilde I_2$ are the first and second invariants of the deviatoric right Cauchy–Green deformation tensor, $\tilde \lambda$ is the deviatoric part of the stretch ratio along the fiber direction, *K* is a bulk modulus, and *J* is the determinant of the deformation gradient tensor. The function *F*_1_ represents the strain energy associated with the isotropic material and is given by:

4 $$F_1\left( {\tilde I_1,\tilde I_2} \right) = C_1\left( {\tilde I_1 - 3} \right) + C_2\left( {\tilde I_2 - 3} \right),$$

where *C*_1_ and *C*_2_ are Mooney–Rivlin material coefficients. $F_2\left( {\tilde \lambda } \right)$ is the strain energy associated with fiber stretch and is described by:

5 $$\begin{array}{l}\tilde \lambda \frac{{\partial F}}{{\partial \lambda }} = 0,\quad \quad \quad \quad \quad \quad \quad \tilde \lambda \le 1;\\ \tilde \lambda \frac{{\partial F}}{{\partial \lambda }} = C_3e^{C_4(\tilde \lambda - 1)} - 1,\quad < \tilde \lambda \le \lambda ^ \ast ;\\ \tilde \lambda \frac{{\partial F}}{{\partial \lambda }} = C_5\tilde \lambda + C_6,\quad \quad \ \quad \tilde \lambda \ge \lambda ^ \ast ;\end{array}$$

where *C*_3_ scales the exponential stresses, *C*_4_ modulates the rate of uncrimping of fibers, λ^*^ is the stretch at which the fibers straighten, and *C*_5_ is the modulus of the straightened fibers^18^. Material coefficients (see Supplementary Fig. 1 caption) were adapted from the literature^16,19,40^.

Tendon geometry was modeled as a cylindrical structure with a 3 mm radius and 60 mm length. The geometry was meshed with 12,960 hexahedral elements. Fibers were set to be initially aligned with the long axis of the tendon. For each simulation, the tendon was fixed at one end and the other end was displaced to induce an axial strain between 0 and 10%. An impulsive transverse displacement (10 µm over 0.1 ms) was introduced at one end of the tendon. Numerical integration of the system equations of motion was used to simulate propagation of the resulting wave along the tissue. Time of arrival of the wave at points 10, 20, and 30 mm from the impulsive input displacement were used to measure wave propagation speed. Simulations were repeated with a ground substance material model that produced a shear elastic modulus of 0.1, 0.2, 0.4, 0.8, and 1.6 MPa. See caption of Supplementary Fig. 1 for full set of parameters used in the simulation. For each case, we compared the tensioned beam model predictions of wave speed (Eq. 2) with the finite element model predictions over the range of strains considered. All simulations were performed in FEBio (www.febio.org).

### Ex vivo testing of porcine flexor tendons

Digital flexor tendon specimens (*N* = 10) were excised from 6–10-month-old porcine feet from approximately 2 cm beyond the distal muscle–tendon junction to the bony distal insertion. Each specimen was wrapped in a normal saline (NS; 9.0 g L^−1^ NaCl)-soaked towel and placed in a freezer in the time between dissection and testing. Tendons were fully thawed in NS prior to mechanical testing. Tendons were secured at each end by waveform grips (G240KSS, Test Resources) in an electrodynamic mechanical test system (Acumen 3, MTS). Specimens were tested in an NS solution contained within a custom-built bath to maintain hydration. A 10 MHz, 38 mm wide linear array transducer (L14-5W/38, BK Utrasound, Richmond, BC, Canada) was affixed to the front panel of the bath and used to image the tendon through an acoustic window. B-Mode ultrasonic imaging (SonixTOUCH Research, BK Utrasound) was initially performed to align the transducer with the longitudinal central axis of the tendon.

### Mechanical tapping device

A tapper was constructed that consisted of a piezoelectric stack (PK4JQP2, Thorlabs) mounted in a 3D-printed housing (Supplementary Fig. 2). The piezoelectric stack provided linear actuation (19.8 μm displacement at 150 V) of a 90 mm long push rod that was positioned to be in contact with the relaxed tendon at a point that was 22 mm from the bottom grip. A signal generator (SDG1025, Siglent) was used to produce a pulse sequence (1 ms pulse, 25 Hz repetition rate) that was amplified by an open-loop piezo controller (MDT694B, Thorlabs) and used to drive the tapper.

### Ultrasonic measurement of tendon motion

A research ultrasound system (SonixTOUCH Research, BK Utrasound) was programmed to collect radiofrequency (RF) data from a single element located in the middle of the transducer array. RF signals were collected at 40 MHz to a depth of 2 cm. With a sound propagation speed of 1560 m s^−1^, the spatial resolution of the signal along the sample line was 19 μm. Sampling from a single element allowed us to sample RF data at an ultra-high frame rate (14,100 frames per second), which was needed to track transverse tendon vibration. A one-dimensional speckle-tracking algorithm was used to track tendon tissue motion from the RF data^41^. At each frame, RF signals were first upsampled by a factor of 4 using a polyphase anti-aliasing filter (resample; MATLAB). We then identified the location of the near and far edges of the tendon based on the amplitude of the RF signal. A series of 0.5 mm long kernels (no overlap) were then placed over the width of the tendon. The frame-to-frame displacements of each kernel were computed by finding the spatial shift that would maximize the normalized cross-correlation of the RF signal within the kernel with the RF signal at the subsequent time step. Cosine interpolation of the normalized cross-correlation functions was used to compute sub-pixel displacements^42^. Kernel velocity was then computed by multiplying the incremental displacement by the frame rate.

### Mechanical testing

Tendons were cyclically stretched from 10 to 300 N at 0.5 Hz, 1.0 Hz, and 2.0 Hz. The tendons were initially pre-conditioned with 10 loading cycles. Following a 7-min rest period, subsequent mechanical testing was performed while the tapper delivered impulsive taps to the tendon. For each data collection trial, the tendons were stretched for 10 consecutive cycles, with ultrasonic RF data, crosshead displacements, and axial forces synchronously collected over the final 5 cycles. The major and minor axes were measured at three locations spaced evenly along the tendon. Average tendon cross-sectional area was then computed based on an elliptical fit of each cross-section. Tendon stresses were calculated by normalizing the axial force to the undeformed cross-sectional area.

### Wave speed measurement

The kernel transverse velocities were band-pass filtered (50–2000 Hz) using a second-order, zero-lag, Butterworth filter to isolate the signal associated with the tapper-induced vibration. For each tap, the transverse vibration frequency, *f*, was determined by performing a fast Fourier transform on the transient velocity trajectory and finding the peak amplitude of the spectrum. Wave speed was then calculated assuming a first mode of vibration, *c *= 2*fL*, where *L* is the distance between the upper and lower grips. A separate collection of transient tendon motion at multiple points along the tendon was performed and confirmed that the standing wave represented the first vibratory mode.

The coefficient of determination (*R*^2^) was used to assess the relationship between squared wave speed and axial tendon stress. The effect of loading rate on the wave speed–stress relationship was assessed in 10 tendons using a one-way repeated-measures analysis of variance on linear regression slopes of squared wave speed versus stress for each loading rate. Stress data were separated into 15 evenly spaced bins, and corresponding mean and s.d. of squared wave speeds were used to visually assess agreement with the wave propagation model (Fig. 1). All calculations were performed in MATLAB.

### Analytical predictions of wave speed

The tensioned beam model (Eq. 2) was used to compute predicted wave speeds based on literature estimates of shear modulus (*μ* = 0.04–1.6 MPa) and shear correction factor^43^ (*k’* *=* 0.9), and empirical measurements of the tendon cross-sectional area (*A* = 22.6 ± 5.2 mm^2^) and estimates of the effective density (*ρ* = 1730 kg m^−3^). The effective density reflects both the measured wet tendon density (1060 kg m^−3^) and added mass effect caused by entrained motion of the surrounding fluid (see supplementary methods).

Since no estimates are available for shear modulus of loaded tendon, we considered a range of shear modulus values (0.04 to 1.6 MPa) based on measurements for ligament under shear deformation^16^. Shear wave elastography has been used to measure wave speeds of approximately 8 m s^−1^ in unloaded porcine digital flexor tendons^44^. If the classical shear wave speed equation $\left( {c^2 = {{\it{\mu }}/{\it{\rho }}} } \right)$ is used, this corresponds to a shear modulus estimate of approximately 0.06 MPa. This shear modulus is at the lower end of the range we considered in our model estimates, as would be expected in a strain-stiffening material such as tendon.

### In vivo testing

Six healthy adults participated in the gait collections. Five subjects (2 female, 3 male; age = 23.2 ± 2.4 years; mass = 73.9 ± 17.0 kg; height = 1.79 ± 0.13 m) underwent Achilles and patellar tendon wave speed collections during treadmill walking. Representative collections were performed on the patellar tendon of one subject (male, 27 years old., 74.4 kg, 1.88 m) during treadmill running at varied cadences, and on the lateral hamstrings of another subject (male, 21 years old, 70.3 kg, 1.73 m) during treadmill running at a range of speeds. All participants provided informed consent prior to testing. The study protocol was approved and overseen by the Institutional Review Board of the University of Wisconsin-Madison.

### Mechanical tapping device

For human subject testing, a low-profile tapper device was constructed that could be securely strapped over the Achilles, patellar, or hamstring tendons. The tapper consisted of an 18.8 mm piezoelectric stack actuator (PK4JQP2, Thorlabs) driving a lever that rotated about a 3 mm stainless steel hinge within a 3D-printed housing (Supplementary Fig. 2). The lever amplified the linear displacement of the actuator (19.8 μm displacement at 150 V) by a factor of 2, resulting in a 39.6 μm displacement at the skin. A signal generator (FY2300A, FeelTech) produced a square wave pulse sequence (0.5–9.5 V, 50% duty cycle, 50 Hz repetition rate) that was amplified by an open-loop piezo controller (MDT694B, Thorlabs) and used to drive the tapper. Transient shear waves were induced in tendon by both extension and retraction of the actuator. In this study we present wave speeds derived only from the extension of the actuator, such that wave speeds were tracked at 50 Hz.

### Ultrasonic measurement of tendon motion

We initially used high frame rate ultrasonic imaging to track Achilles tendon motion in the human subject experiments. A 20 mm wide linear array transducer (HST15-8/20, BK Utrasound) was secured over the Achilles tendon. A tapper (Fig. 2) was positioned over the tendon below the transducer and approximately 20 mm superior to the proximal edge of the calcaneus. B-Mode ultrasonic imaging was initially used to align the transducer with the longitudinal central axis of the tendon. The ultrasound system (SonixTOUCH Research, BK Utrasound) was programmed to collect RF signals at 40 MHz from two elements separated by 9.3 mm. The first element was located approximately 25 mm from the tapper. With an imaging depth of 20 mm, the RF data were sampled at a frame rate of 14,100 frames per second. For each element, a one-dimensional speckle-tracking algorithm was used to compute kernel velocities in a manner equivalent to that used in the ex vivo testing.

### Accelerometer-based measurement of tendon motion

We measured transverse motion of the tendon at two locations using single-axis accelerometers (Model 352C23, PCB Piezotronics) with 0.5 mV m^−1^ s^2^ sensitivity. The miniature (8.6 × 4.1 × 2.8 mm, 0.2 g) accelerometers were secured in tight-fitting, 3D-printed housings to facilitate incorporation into an elastic strap (Fig. 3). The first accelerometer was positioned approximately 10 mm from the tapper. Inter-accelerometer distances (center-to-center) were fixed at 10 mm and 8 mm for the Achilles and patellar tendons, respectively. Inter-accelerometer distance for the patellar tendon was decreased slightly due to the relatively limited tendon length available for measurement in some subjects. Accelerometer signals were amplified (Model 480B21, PCB Piezotronics) and sampled at 50 kHz using LabVIEW (National Instruments).

### Wave speed calculation using accelerometer data

Accelerometer data were band-pass filtered (150–1000 Hz) using a second-order, zero-lag, Butterworth filter to isolate the signal associated with induced shear waves. Data were segmented into 1000-frame (20 ms) windows starting at each leading edge of the tapper excitation signal (i.e., tapper extension). Within each window, data from each accelerometer were mean-centered, normalized to the maximum absolute magnitude, squared to enhance peak magnitudes, and multiplied by the sign of the original measurement to preserve the wave shape. Subsequently, the inter-accelerometer lag in wave arrival time was determined by finding the lag that maximized the cross-correlation between each windowed accelerometer signal. Cosine interpolation of the normalized cross-correlation functions was used to estimate the location of peak cross-correlation with sub-frame accuracy^42^. Finally, wave speed was calculated by dividing the fixed inter-accelerometer distance by the computed inter-accelerometer time lag. All calculations were performed in MATLAB (MathWorks).

### Isometric testing

Subjects performed isometric knee extension and ankle plantarflexion exertions cyclically at three rates (0.25, 0.50, 1.00 Hz), guided by an auditory metronome. Subjects were asked to generate near-maximal force, but there was no feedback provided on effort level. For the knee extension task, subjects were seated on the edge of a bench with their knee flexed at 90° (Supplementary Fig. 4). A cuff around the ankle was attached to a fixed load cell (LCM300, Futek) via a cable. Knee extension forces were sampled at 50 kHz in synchrony with tendon accelerations.

For the ankle plantarflexion task, subjects were seated in a chair with their ankle dorsiflexed 10° and their knee flexed at 20° (Supplementary Fig. 4). Subjects pushed against a force plate (BalanceCheck, Bertec Corp.) with a cylinder adhered to the force plate and positioned under the head of their first metatarsal to control the point of load application. Ankle plantarflexion forces were recorded at 2000 Hz, and synchronized post hoc with tendon acceleration signals by simultaneously recording an impulsive impact event using both the force plate and a third accelerometer, which was sampled synchronously with the tensiometer accelerometers (50 kHz).

### Walking gait testing

Subjects walked on an instrumented split-belt treadmill (Bertec Corp.) at five fixed speeds (0.75, 1.00, 1.25, 1.50, 1.75 m s^−1^). Ground reaction force (GRF) data were collected at 2000 Hz. Kinematic data were collected at 200 Hz using an eight-camera passive motion capture system (Eagle, Motion Analysis). Lower body (right leg and pelvis) motion was tracked using a set of 10 anatomical markers placed at the right and left anterior superior and posterior superior iliac spines, the sacrum, the right lateral femoral epicondyle, tibial tuberosity, lateral malleolus, calcaneus, and fifth metatarsal-phalangeal joint, and 7 markers on the right thigh and shank. Wireless surface EMG sensors (Trigno, Delsys) were secured over the right vastus lateralis, biceps femoris, medial gastrocnemius, soleus, and tibialis anterior muscles. Sensors were placed according to SENIAM guidelines (www.seniam.org), and EMG signals were recorded at 2000 Hz.

### Running gait testing

Two subjects were tested during running on a treadmill. For the step cadence example (Fig. 5a), a tensiometer was secured over the patellar tendon. The subject first warmed up on a treadmill at his preferred speed and cadence, which were recorded. An audible metronome was then used to guide three different step cadences (preferred, ±10%) while the treadmill speed was maintained. For each cadence, patellar tendon wave speeds were collected over a minimum of 10 consecutive strides. For the running speed example (Fig. 5b), the tensiometer was secured over the distal tendon of the lateral hamstrings (biceps femoris). Running speed was varied between 2.68 and 8.05 m s^−1^. At each speed, lateral hamstring wave speed data were collected for a minimum of 5 consecutive strides. Running bouts at each speed were kept to under 10 strides and a minimum of 2 min rest between trials was used to mitigate fatigue effects. The authors affirm that the research participant appearing in the photographs included in Fig. 5 provided informed consent for their publication.

### Analysis

For isometric testing, net knee extension and ankle plantarflexion torques were calculated based on the fixed distance between the load application points and the inter-epicondylar axis of the knee and inter-malleolar axis of the ankle, respectively. Joint torque and wave speed data were low-pass filtered at 20 Hz using a second-order, zero-lag, Butterworth filter. For each subject, the coefficient of determination (*R*^2^) was used to assess the linear relationship between squared wave speed and joint torque. For each loading rate, stress data were separated into eight evenly spaced bins. Mean and s.d. of squared wave speeds corresponding to each bin were used alongside linear fits to visually assess linearity (Fig. 3d).

For walking gait testing, marker trajectories and GRF data were low-pass filtered with cut-off frequencies of 6 Hz and 50 Hz, respectively. EMG data were band-pass filtered (20-450 Hz, Trigno, Delsys) and full-wave rectified. Ankle and knee torques were computed using previously described inverse kinematics and inverse dynamics techniques^45^. Both torque and wave speed data were low-pass filtered at 20 Hz using a second-order, zero-lag, Butterworth filter. Ankle torque and squared Achilles tendon wave speed data were ensemble averaged within each subject across multiple strides.

For running gait testing, strides were delineated by using the distinct acceleration spikes seen at heel strike. Tendon wave speed data were then ensemble averaged within each subject across multiple strides.

### Code availability

Post hoc analyses of experimental data were performed using code written in MATLAB (R2016b, MathWorks), essential portions of which are available from the corresponding author on reasonable request. Finite element models were created and solved using Finite Elements for Biomechanics (FEBio v. 2.5.2, https://febio.org/), and are available from the corresponding author upon request.

### Data availability

Datasets generated and/or analyzed during the current study are available from the corresponding author on reasonable request.

## Electronic supplementary material

Supplementary Information
