# Supplementary material for: Gauging force by tapping tendons
Source: Nat Commun. 2018 Apr 23;9:1592. doi: 10.1038/s41467-018-03797-6 (PMC5913259; doi:10.1038/s41467-018-03797-6)
Supplement: Supplementary file 1 — Supplementary Information [file 41467_2018_3797_MOESM1_ESM.pdf]

Supplementary Information:

Gauging Force by Tapping Tendons

Martin et al.

Supplementary Figures

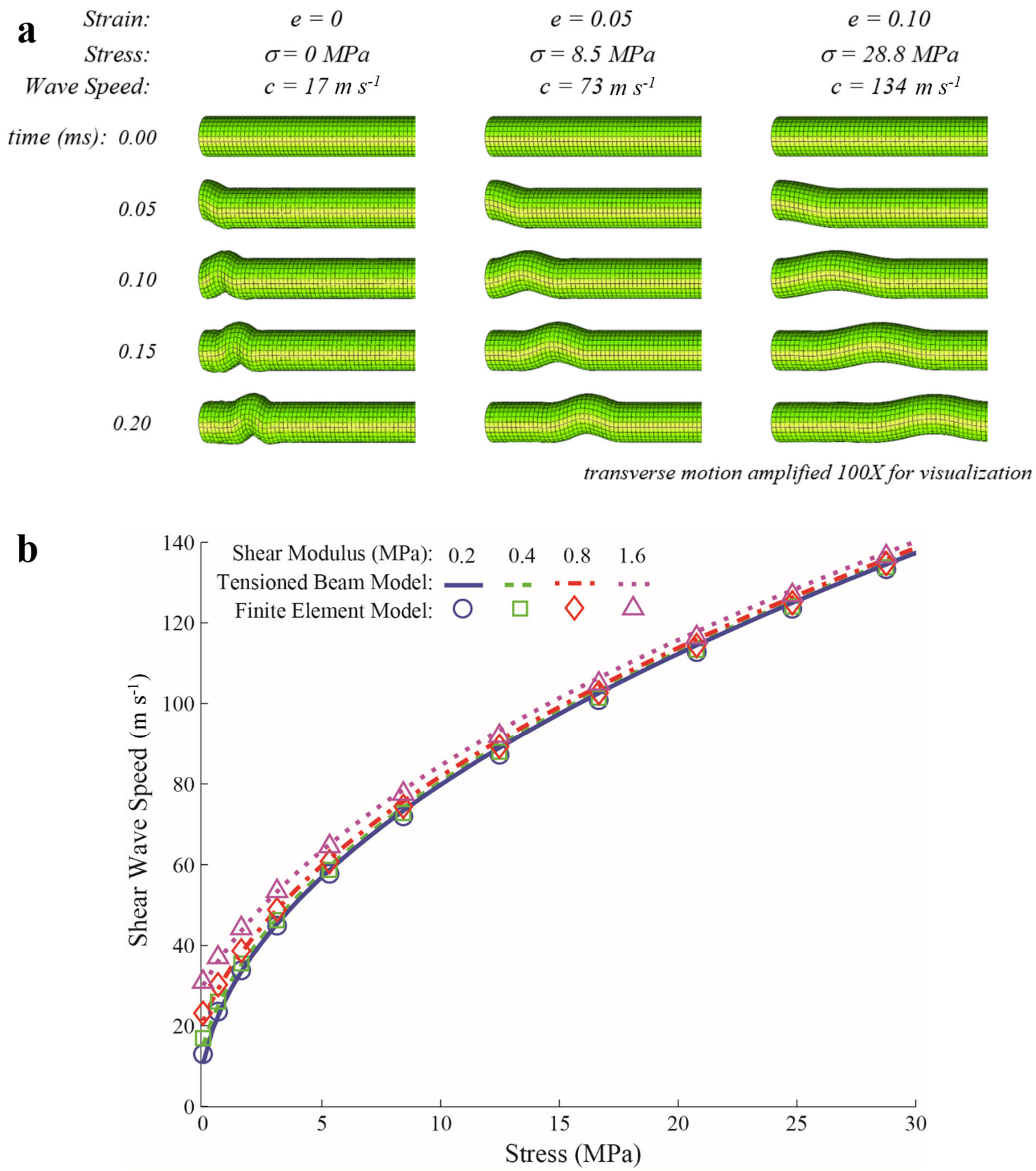

Supplementary Figure 1 | Finite Element Modeling. a. A nonlinear finite element (FE) model was used to simulate wave propagation in relaxed and stretched tendons. Tendon behavior was described by a transversely isotropic hyperelastic material. The structurally motivated material model represents nonlinear elastic fibers interacting with an isotropic matrix described by a Mooney-Rivlin formulation (see Methods). The material parameters were: $C_1 = 0.2$ MPa, $C_2 = 0$, $C_3 = 1$ MPa, $C_4 = 50$, $C_5 = 600$ MPa, $\lambda^* = 1.05$, density $\rho = 1600$ kg m^{-3} and bulk modulus $K = 200$ MPa. Variables: $e \equiv$ axial strain; $\sigma \equiv$ axial stress; $c \equiv$ wave speed. **b.** FE model simulated wave speeds agree well with predictions from the tensioned beam model. The dominant effect of stress on wave speed was the result of aligned fibers producing a high axial stiffness when taut, while the matrix had a relatively low shear modulus ($= 2C_1$). All simulations were performed in FEBio (www.febio.org).

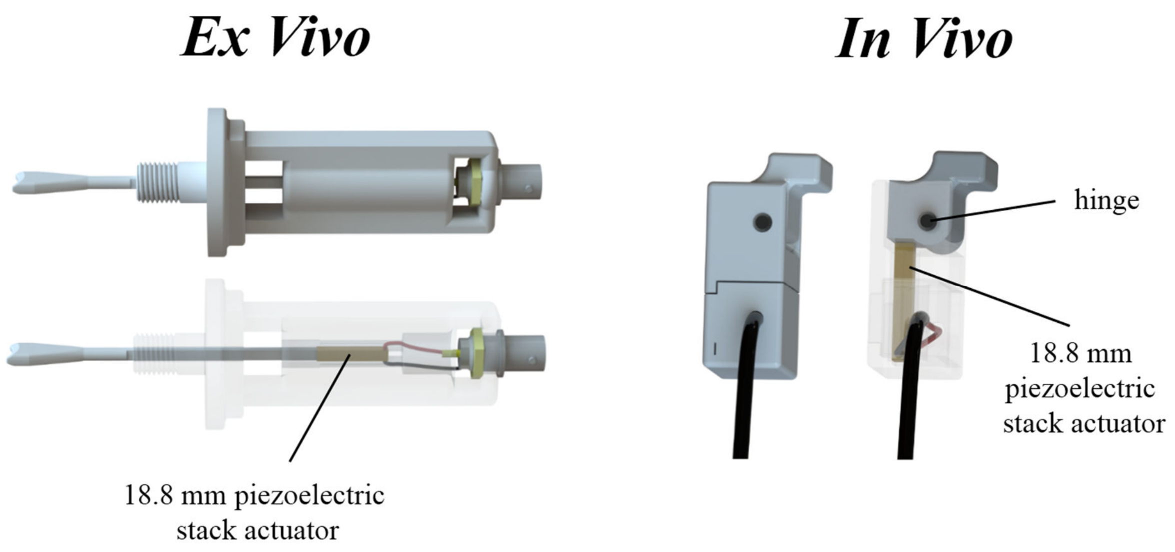

Supplementary Figure 2 | Tapper Devices. Custom "tapper" devices were designed to induce shear waves in tendon during *ex vivo* (left) and *in vivo* (right) experiments. Each tapper consisted of a 3D-printed housing and an 18.8mm piezoelectric stack actuator (Thorlabs). For the *ex vivo* tapper (left), the piezo stack pushed a 90 mm long pushrod transversely against the tendon at a rate of 25 Hz (1 ms pulse) with a 19.8 µm stroke length. The *in vivo* tapper (right) utilized a hinge mechanism to i) change the direction of motion, thereby reducing the tapper's projection from the surface of the skin and ii) to mechanically amplify (2:1) transverse tendon displacement (39.6 µm stroke). This tapper was actuated at 50 Hz (10 ms pulse).

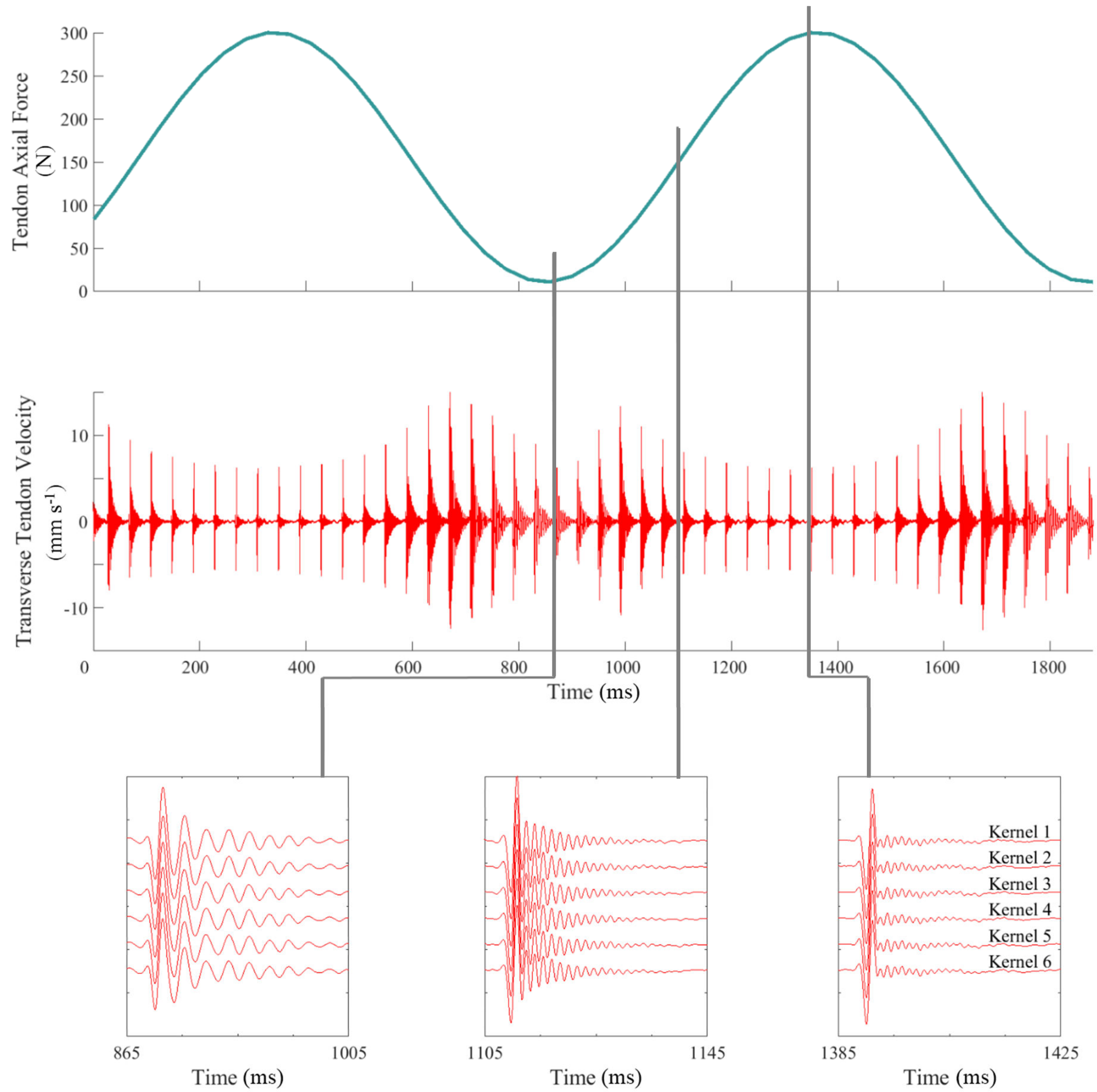

Supplementary Figure 3 | Load dependence of *ex vivo* tendon vibration frequency. During *ex vivo* tensile tests, each porcine tendon was clamped in a mechanical testing machine and sinusoidally loaded in the axial direction (Top) while transverse vibrations were intermittently induced (Middle). Speckle-tracking of ultrasonic radiofrequency data was used to track velocity of a series of non-overlapping 0.5 mm kernels across the width of the tendon. For each of the taps, the kernels exhibited similar underdamped standing waves suggesting bulk tendon motion was induced (Bottom). Note the systematic increase in tendon vibration frequency with axial force (Bottom, Left to Right). Wave speed c was computed knowing the oscillation frequency, f, and the length of the porcine tendon, L ($c = 2fL$).

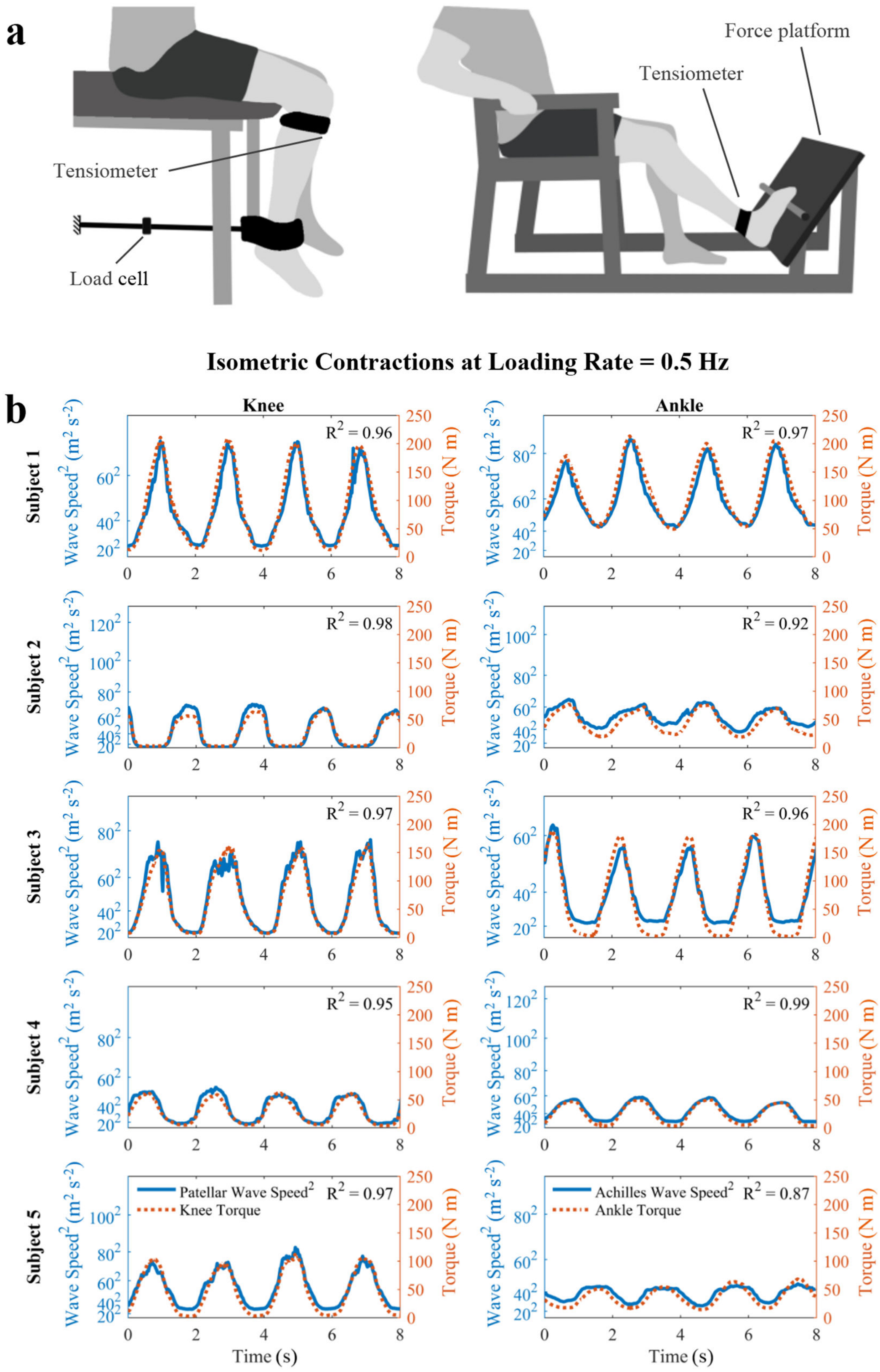
a
Force platform
Tensiometer
Tensiometer
Load cell
Isometric Contractions at Loading Rate = 0.5 Hz
b
Knee
Ankle
Subject 1
Subject 2
Subject 3
Subject 4
Subject 5
Wave Speed^2 (m^2 s^{-2})
Torque (N m)
R^2 = 0.96
R^2 = 0.97
R^2 = 0.98
R^2 = 0.92
R^2 = 0.97
R^2 = 0.96
R^2 = 0.95
R^2 = 0.99
R^2 = 0.97
R^2 = 0.87
Patellar Wave Speed^2
Knee Torque
Achilles Wave Speed^2
Ankle Torque
Time (s)

Supplementary Figure 4 | Isometric testing. a. Isometric testing setups for knee extension and ankle plantarflexion. **b.** The patellar and Achilles tendon wave speeds varied in phase with isometric knee and ankle torque, respectively, during isometric contractions. Coefficients of determination (R^2) between wave speed^2 and torque were high for all subjects, though the slope of the linear relationship (Achilles: 36.1±17.9 $m^2\ s^{-2}\ N^{-1}\ m^{-1}$; Patellar: 45.7±20.4 $m^2\ s^{-2}\ N^{-1}\ m^{-1}$) varied between subjects and between tendons. This variability may reflect inherent differences in tendon cross-sectional area and moment arms which modulate the relationship between tendon stress and torque. Average within-subject variability was low (Achilles: ±7.2 $m^2\ s^{-2}\ N^{-1}\ m^{-1}$; Patellar: ±4.0 $m^2\ s^{-2}\ N^{-1}\ m^{-1}$), indicating that loading rate did not strongly affect the wave speed-joint torque relationship.

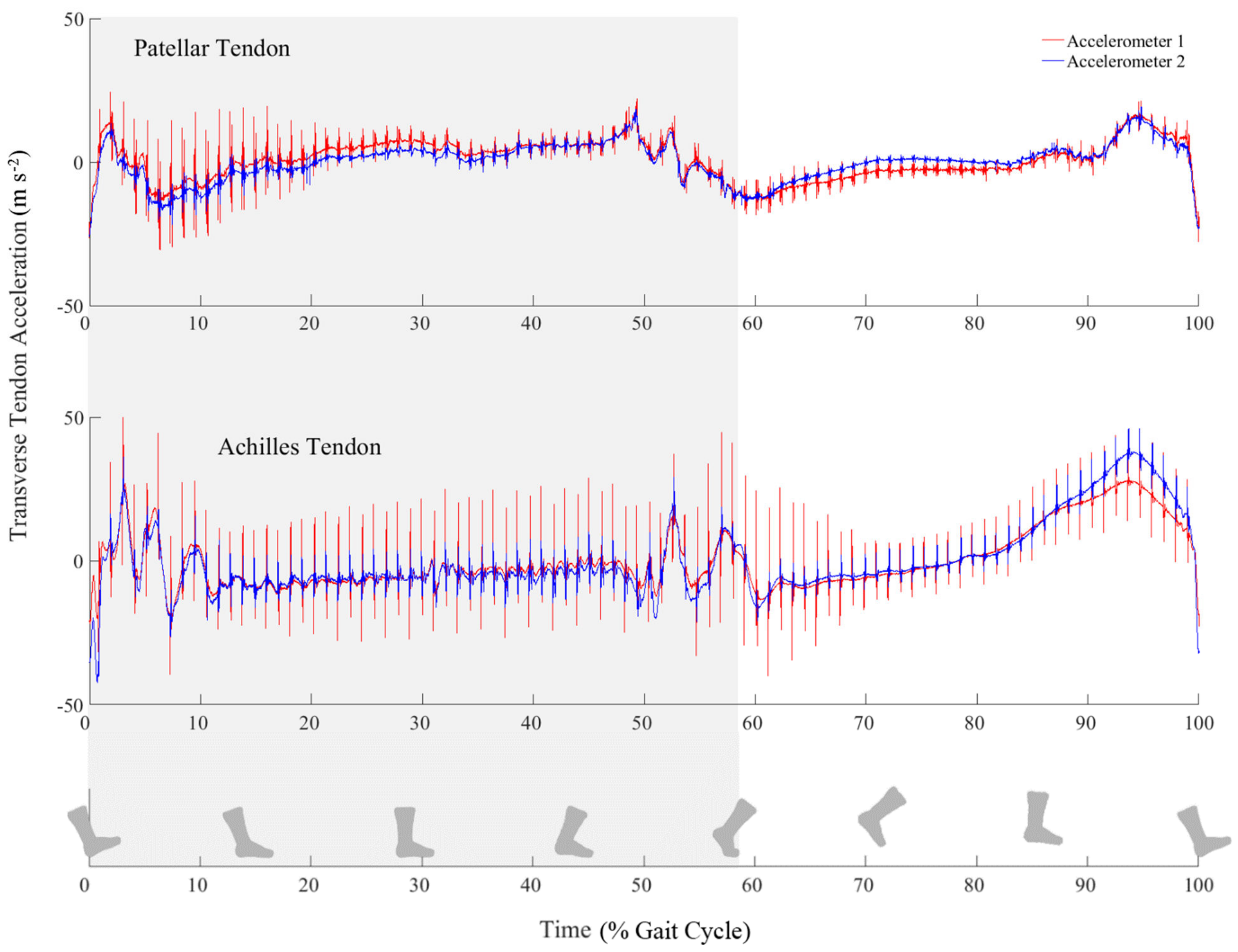

Supplementary Figure 5 | Raw Achilles tendon accelerations during gait. Accelerometers were secured over both patellar (Top) and Achilles (Bottom) tendons, and used to measure tendon transverse acceleration at two locations (red: near accelerometer, blue: far accelerometer). The shaded region indicates stance phase, which extends from heel strike (0%) to toe-off (58% gait cycle). Swing phase extends from 58% to 100% of the gait cycle. Each high frequency peak corresponds with a shear wave that was induced by the tapper device. Time delay between wave arrival at the two accelerometers was used to compute wave propagation speed.

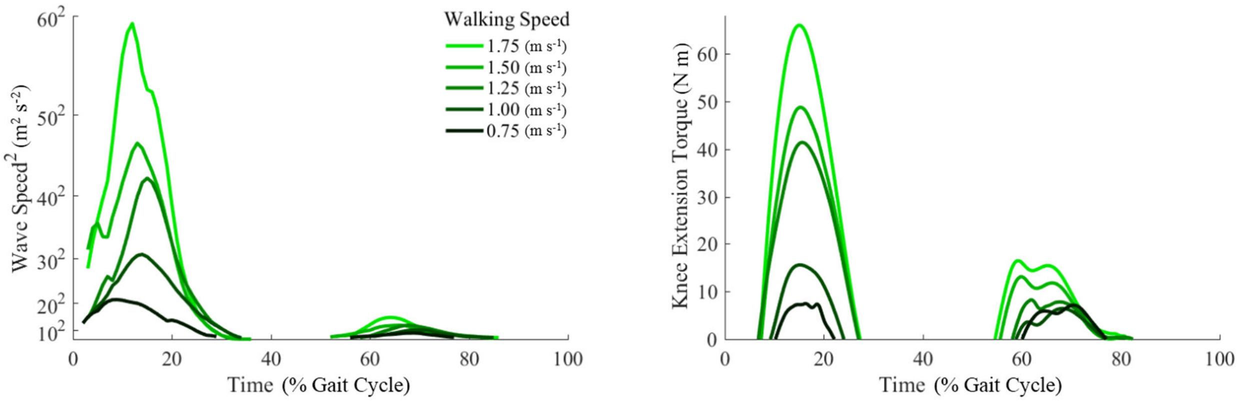

Supplementary Figure 6 | Patellar tendon wave speed during gait: speed effects. Patellar tendon wave speeds (left) and knee extension torque (right) were measured *in vivo* throughout the gait cycle at five walking speeds (0.75 – 1.75 m s^{-1}). Results are presented here for a single subject (subject 3), averaged across multiple strides. Both the wave speed and knee extension torque exhibit a primary peak during the load acceptance phase (~15 % gait cycle). A smaller second peak in both metrics is seen slightly after toe-off (~65 % gait cycle). Similar speed-dependent increases in squared wave speed and knee torque are observed, supporting the use of tendon wave speed as indicator of tissue loading. Regions where wave speed is not plotted reflect phases of gait when the patellar tendon went relatively slack (knee extension torque < 0) and tapping did not induce sufficient accelerometer signals to track wave speeds.

Supplementary Tables

Supplementary Table 1 | Variable Definitions for Supplementary Methods

Variable	Definition
$\boldsymbol{x}$	Lateral position on the beam
$\boldsymbol{t}$	Time
$\boldsymbol{A}$	Cross-sectional area
$\boldsymbol{b}$	Minor dimension of elliptical cross-section
$\boldsymbol{a}$	Major dimension of elliptical cross-section
$\boldsymbol{I}$	Bending moment of inertia
$\boldsymbol{k'}$	Shear correction factor
$\boldsymbol{\rho}$	Density (effective density in the main text)
$\boldsymbol{E}$	Axial elastic modulus
$\boldsymbol{\mu}$	Transverse plane tangential shear modulus
$\boldsymbol{L}$	Length
$\boldsymbol{\gamma(x,t)}$	Shear strain
$\boldsymbol{\tau(x,t)}$	Shear stress
$\boldsymbol{T(x)}$	Axial tension
$\boldsymbol{\sigma(x)}$	Axial stress
$\boldsymbol{w(x,t)}$	Lateral beam deflection
$\boldsymbol{W}$	Magnitude of lateral deflection
$\boldsymbol{\theta(x,t)}$	Beam rotation due to bending
$\boldsymbol{\Theta}$	Magnitude of rotation
$\boldsymbol{q(x,t)}$	Applied lateral force density
$\boldsymbol{Q}$	Magnitude of applied lateral force
$\boldsymbol{f}$	Vibration frequency [Hz]
$\boldsymbol{\omega}$	Vibration frequency [rad s^{-1}]
$\boldsymbol{K(\omega)}$	Wavenumber
$\boldsymbol{\mathcal{P}(t)}$	Strain energy
$\boldsymbol{\mathcal{T}(t)}$	Kinetic energy
$\boldsymbol{\mathcal{W}(t)}$	Imposed work
$\boldsymbol{m_{eff}}$	Effective mass
$\boldsymbol{\rho_{eff}}$	Effective density
$\boldsymbol{\rho_t}$	Wet tendon density
$\boldsymbol{\rho_f}$	Fluid density
$\boldsymbol{\Gamma_r(\omega)}$	Real part of the hydrodynamic function for a beam of circular cross-section

Supplementary Table 2 | Parameter Values

Parameter	Value
$\boldsymbol{\mu}$	$0.04 - 1.6\ MPa$
$\boldsymbol{k'}$	0.9
$\boldsymbol{\rho_t}$	$1060\ kg\ m^{-3}$
$\boldsymbol{\rho_f}$	$1000\ kg\ m^{-3}$
$\boldsymbol{\rho_{eff}\ (\rho)}$	$1730\ kg\ m^{-3}$
$\boldsymbol{\Gamma_r}$	1.07

Supplementary Methods

Full Derivation of Equation for Wave Propagation Speed

We modeled tendons as tensioned Timoshenko beams[1,2], as briefly reviewed here. We consider an elastic beam subject to shear, bending, and extensional deformation. The associated strain energy is:

$$\mathcal{P} = \frac{1}{2}\int_0^L EI\left(\frac{\partial\theta}{\partial x}\right)^2 dx + \frac{1}{2}\int_0^L k'\mu A\gamma^2 dx + \frac{T}{2}\int_0^L \left(\frac{\partial w}{\partial x}\right)^2 dx \qquad (1)$$

Noting that the slope of the beam is comprised of the shear and bending deflections:

$$\frac{\partial w}{\partial x} = \gamma + \theta \qquad (2)$$

we can use this to eliminate shear strain and rewrite Eqn. 1 as:

$$\mathcal{P} = \frac{1}{2}\int_0^L EI\left(\frac{\partial\theta}{\partial x}\right)^2 dx + \frac{1}{2}\int_0^L k'\mu A\left(\frac{\partial w}{\partial x} - \theta\right)^2 dx + \frac{T}{2}\int_0^L \left(\frac{\partial w}{\partial x}\right)^2 dx \qquad (3)$$

Additionally, the instantaneous kinetic energy of the beam due to shear and bending is:

$$\mathcal{T} = \frac{1}{2}\int_0^L \rho A\left(\frac{\partial w}{\partial t}\right)^2 dx + \frac{1}{2}\int_0^L \rho I\left(\frac{\partial\theta}{\partial t}\right)^2 dx \qquad (4)$$

and the work imposed on the beam by lateral forces is:

$$\mathcal{W} = \int_0^L w(x,t)q(x)dx \qquad (5)$$

While ignoring initial and boundary conditions, application of Hamilton's principle gives:

$$\int_{t_1}^{t_2} (\delta\mathcal{T} - \delta\mathcal{P} + \delta\mathcal{W})dt = 0 \qquad (6)$$

Shear and bending deformations occur independently, so we must consider variations with respect to both w and θ:

$$\int_{t_1}^{t_2}\int_0^L \left[\rho A\frac{\partial w}{\partial t}\delta\left(\frac{\partial w}{\partial t}\right) - k'\mu A\left(\frac{\partial w}{\partial x} - \theta\right)\delta\left(\frac{\partial w}{\partial x}\right) - T\frac{\partial w}{\partial x}\delta\left(\frac{\partial w}{\partial x}\right) + q\delta w\right] dx\, dt = 0 \qquad (7)$$

$$\int_{t_1}^{t_2}\int_0^L \left[\rho I\frac{\partial\theta}{\partial t}\delta\left(\frac{\partial\theta}{\partial t}\right) - EI\frac{\partial\theta}{\partial x}\delta\left(\frac{\partial\theta}{\partial x}\right) + k'\mu A\left(\frac{\partial w}{\partial x} - \theta\right)\delta\theta\right] dx\, dt = 0 \qquad (8)$$

Recognizing that virtual displacements, δw, and virtual rotations, $\delta\theta$, can be taken separately, and performing the appropriate integration by parts, the governing equations can be obtained from Eqns. 7 and 8:

$$-\rho A \frac{\partial^2 w}{\partial t^2} + (k'\mu A + T)\frac{\partial^2 w}{\partial x^2} - k'\mu A \frac{\partial \theta}{\partial x} + q = 0 \tag{9}$$

$$-\rho I \frac{\partial^2 \theta}{\partial t^2} + EI \frac{\partial^2 \theta}{\partial x^2} + k'\mu A\left(\frac{\partial w}{\partial x} - \theta\right) = 0 \tag{10}$$

We can then study wave propagation under the assumption that the motion is harmonic in time and space:

$$w = \Re\{We^{i(\omega t+Kx)}\}, \qquad \theta = \Re\{\Theta e^{i(\omega t+Kx)}\}, \qquad q = \Re\{Qe^{i(\omega t+Kx)}\} \tag{11}$$

Substituting Eqns. 11 into Eqns. 9 and 10 gives:

$$\omega^2 \rho A W - K^2(k'\mu A + T)W - iKk'\mu A\Theta + \mathrm{Q} = 0 \tag{12}$$

$$\omega^2 \rho I\Theta - K^2 EI\Theta + k'\mu A(\mathrm{iKW} - \Theta) = 0 \tag{13}$$

To determine the relative magnitude of bending versus shear deformation, we can rearrange Eqn. 13 to obtain:

$$\frac{\Theta}{W} = \frac{-iKk'\mu A}{\omega^2 \rho I - K^2 EI - k'\mu A} \tag{14}$$

Eqn. 14 shows that the rotations will be small relative to shear deformation for cases of high bending stiffness (i.e., large EI) or high vibration frequency. In these cases, the Θ term in Eqn. 12, which links shear deformation to bending, can be ignored, and Eqn. 12 is reduced to:

$$\omega^2 \rho A W - K^2(k'\mu A + T)W + \mathrm{Q} = 0 \tag{15}$$

Now, in the case of free vibration, where Q is zero, wave speed can be calculated from Eqn. 15 as:

$$c^2 = \frac{\omega^2}{K^2} = \frac{k'\mu A + T}{\rho A} \tag{16}$$

Factoring out area, Eqn. 16 becomes:

$$c^2 = \frac{k'\mu + \sigma}{\rho} \tag{17}$$

Simplified Derivation for Negligible Bending Case

The simplified derivation presented below is meant to be easier for readers to follow, but note that it does not convey the effects of the approximations that have been made in the full derivation. If we assume from the start that bending deformation is negligible, the same expression for wave speed may be derived much more simply. In this case, we begin by writing a differential force balance for a tensioned beam subject to shear deformations and zero lateral loading:

$$\rho A \frac{\partial^2 w}{\partial t^2} = \frac{\partial(k' A \tau)}{\partial x} + T \frac{\partial^2 w}{\partial x^2} \tag{18}$$

This is a second-order differential equation (in contrast with the Eqns. 12 and 13, which are fourth order in the displacement variable), and so it admits solutions that are non-dispersive shear waves. Using Eqn. 2, we can write the shear stress as:

$$\tau = \mu\gamma = \mu \frac{\partial w}{\partial x} \tag{19}$$

such that Eqn. 18 can be written as:

$$\rho A \frac{\partial^2 w}{\partial t^2} = k' A \mu \frac{\partial^2 w}{\partial x^2} + T \frac{\partial^2 w}{\partial x^2} \tag{20}$$

Combining terms and factoring out area then gives:

$$\rho \frac{\partial^2 w}{\partial t^2} = [k'\mu + \sigma] \frac{\partial^2 w}{\partial x^2} \tag{21}$$

Now, if we assume an arbitrary wave function for shear deformation:

$$w = f(x - ct) \tag{22}$$

we can take the necessary derivatives and write Eqn. 21 as:

$$\rho c^2 f'' = [k'\mu + \sigma] f'' \tag{23}$$

Thus, the previously derived expression for wave speed can again be obtained:

$$c^2 = \frac{k'\mu + \sigma}{\rho} \tag{17}$$

Effective Density

Since the tendon is immersed in fluid, we must account for the increase in effective tendon mass caused by the entrained motion of fluid surrounding the tendon. The problem of a beam vibrating in viscous fluid has been previously solved for applications in atomic force microscopy[3,4], and those results are applied here. Allen and colleagues' definition of effective mass[4] can be rewritten for a beam with ellipsoidal cross-section as follows:

$$\frac{m_{eff}}{L} = \rho_t A + \rho_f \left(\frac{\pi b^2}{4}\right) \Gamma_{\mathrm{r}}(\omega) \tag{24}$$

where the dominant length scale for hydrodynamic flow is the width of the beam perpendicular to the direction of motion[3]. Since we require in our *ex vivo* experiments that the minor dimension of the tendon will be oriented perpendicular to the direction of motion, b can be taken as the minor dimension. Dividing through by area and assuming an elliptical cross-section (with diameters a and b) in Eqn. 24 then gives an effective density:

$$\rho_{eff} = \rho_t + \rho_f \left(\frac{b}{a}\right) \Gamma_{\mathrm{r}}(\omega) \tag{25}$$

The real part of the hydrodynamic function, $\Gamma_{\mathrm{r}}(\omega)$, describes the mass-like effect of the fluid. In a fluid bath that extends infinitely in all directions, the net effect of the fluid is to increase the effective density of the cylinder by a factor, Γ_{r}. The hydrodynamic function is generally not very sensitive to the geometry – for example the hydrodynamic function for a circular cylinder is not that different from that for rectangular beam with infinitesimal thickness (where thickness is measured perpendicular to the direction of motion)[3,5]. Our ellipsoidal cylinder can be reasonably assumed to fall between these two cases, and thus, we employ the hydrodynamic function for a circular cross-section. Using a wet tendon density of 1060 kg m^{-3}, and a fluid density of 1000 kg m^{-3}, the real part of the hydrodynamic function varies between 1.04 and 1.10 over the observed range of vibration frequencies (~150-900 Hz). For simplicity, we used a constant value of 1.07

for the calculations reported in this paper.

For the case of *in vivo* tendons, surrounding tissue likely contributes to the effective tendon mass. In that case there is fluid entrained, and also some elastic tissue, and so the added mass will be governed by a combination of fluid dynamic and elastic forces, rather than fluid dynamics alone. This more complicated case requires further study.

Supplementary References

1. Timoshenko, P. S. P. LXVI. On the correction for shear of the differential equation for transverse vibrations of prismatic bars. *Philos. Mag. Ser. 6* **41,** 744–746 (1921).

2. Timoshenko, P. S. P. X. On the transverse vibrations of bars of uniform cross-section. *Philos. Mag. Ser. 6* **43,** 125–131 (1922).

3. Sader, J. E. Frequency response of cantilever beams immersed in viscous fluids with applications to the atomic force microscope. *J. Appl. Phys.* **84,** 64–76 (1998).

4. Allen, M. S., Sumali, H. & Penegor, P. C. DMCMN: Experimental/Analytical Evaluation of the Effect of Tip Mass on Atomic Force Microscope Cantilever Calibration. *J. Dyn. Syst. Meas. Control* **131,** 064501-064501-10 (2009).

5. Tuck, E. O. Calculation of unsteady flows due to small motions of cylinders in a viscous fluid. *J. Eng. Math.* **3,** 29–44 (1969).
